# Unravelling the Hydration Barrier of Lignin Oleate Nanoparticles for Acid‐ and Base‐Catalyzed Functionalization in Dispersion State

**DOI:** 10.1002/anie.202106743

**Published:** 2021-08-03

**Authors:** Adrian Moreno, Jinrong Liu, Robin Gueret, Seyed Ehsan Hadi, Lennart Bergström, Adam Slabon, Mika H. Sipponen

**Affiliations:** ^1^ Department of Materials and Environmental Chemistry Stockholm University Svante Arrhenius väg 16C 10691 Stockholm Sweden

**Keywords:** biopolymers, colloids, lignin, nanoparticles, organic polymers

## Abstract

Lignin nanoparticles (LNPs) are promising renewable nanomaterials with applications ranging from biomedicine to water purification. However, the instability of LNPs under acidic and basic conditions severely limits their functionalization for improved performance. Here, we show that controlling the degree of esterification can significantly improve the stability of lignin oleate nanoparticles (OLNPs) in acidic and basic aqueous dispersions. The high stability of OLNPs is attributed to the alkyl chains accumulated in the shell of the particle, which delays protonation/deprotonation of carboxylic acid and phenolic hydroxyl groups. Owing to the enhanced stability, acid‐ and base‐catalyzed functionalization of OLNPs at pH 2.0 and pH 12.0 via oxirane ring‐opening reactions were successfully performed. We also demonstrated these new functionalized particles as efficient pH‐switchable dye adsorbents and anticorrosive particulate coatings.

## Graphical abstract

Lignin oleate nanoparticles (OLNPs) are demonstrated for functionalization in aqueous dispersion under acidic (pH 2) and basic (pH 12) conditions owing to the hydration barrier effect provided by oleic acid chains in the shells of OLNPs. Covalent functionalization of these nanoparticles using robust chemistries opens new avenues such as functional anticorrosive coatings and pH‐switchable adsorbents for removal of organic dyes from wastewater.

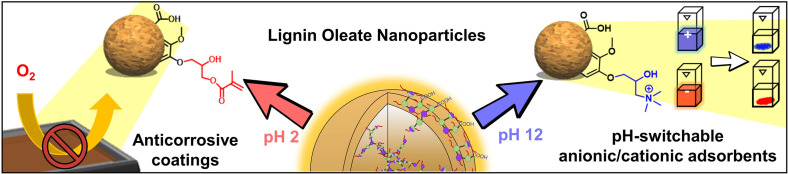

## Introduction

Synthetic polymeric nanoparticles (SPNs) are used in many technological applications owing to their tunable surface chemistry that allows interactions with solid, liquid, and gas interfaces.[^1^, ^2^, ^3^] However, SNPs in the form of latexes and emulsions are invariably made from fossil resources and often display poor biodegradability. There is thus an obvious need for the development of low‐cost and tunable organic nanoparticles that are produced from renewable and biodegradable biomass resources to substitute conventional fossil‐derived nanomaterials.

Lignin appears as a plausible candidate with respect to the above criteria.[^4^, ^5^, ^6^] It is the main component of plant biomass that is removed from cellulose in the chemical pulping processes, making this plant polyphenol abundantly available. However, lignin is currently largely destined for combustion, representing a missed opportunity for functional nanomaterials.[^7^, ^8^, ^9^, ^10^, ^11^, ^12^, ^13^] The ongoing paradigm shift towards lignin‐based advanced engineered materials is largely based on the superior properties of lignin nanoparticles (LNPs), which have emerged as a thriving research field in recent years.[^7^, ^14^, ^15^, ^16^, ^17^]

In contrast to bulk lignin, LNPs resist aggregation in aqueous dispersions (pH 3–9) owing to their sub‐micrometer size and electrostatic repulsion between ionized carboxylic acid groups on their surfaces.^[17]^ This anionic surface charge together with a well‐defined spherical shape and a large surface area to mass ratio make them viable candidates for physical modification via adsorption of positively charged compounds such as enzymes or polymers.[^18^, ^19^] These features of LNPs have contributed to the sprouting of applications in many different areas and materials such as biomedicine,[^8^, ^20^, ^21^, ^22^, ^23^, ^24^] water purification,^[25]^ composites,[^26^, ^27^, ^28^, ^29^, ^30^] biocatalysis,[^31^, ^32^, ^33^] and Pickering emulsions.[^27^, ^34^, ^35^] However, one of the main limitations of LNPs arises from their dissolution in basic (pH>10) and aggregation in acidic (pH<2.5) aqueous solutions, which restricts their functionalization and potential end‐uses.[^36^, ^37^, ^38^, ^39^, ^40^, ^41^] To overcome these shortcomings, stabilization of LNPs via covalent internal cross‐linking has been suggested to overcome solvent instability.[^42^, ^43^, ^44^] However, robust methods for the production of stabilized particles that enable functionalization of LNPs under acidic and basic conditions are still lacking. In this context, development of functionalization routes in the dispersion state is of central importance for extending the range of applications of LNPs to, e.g., coatings and adsorbents.

**Figure 1:**
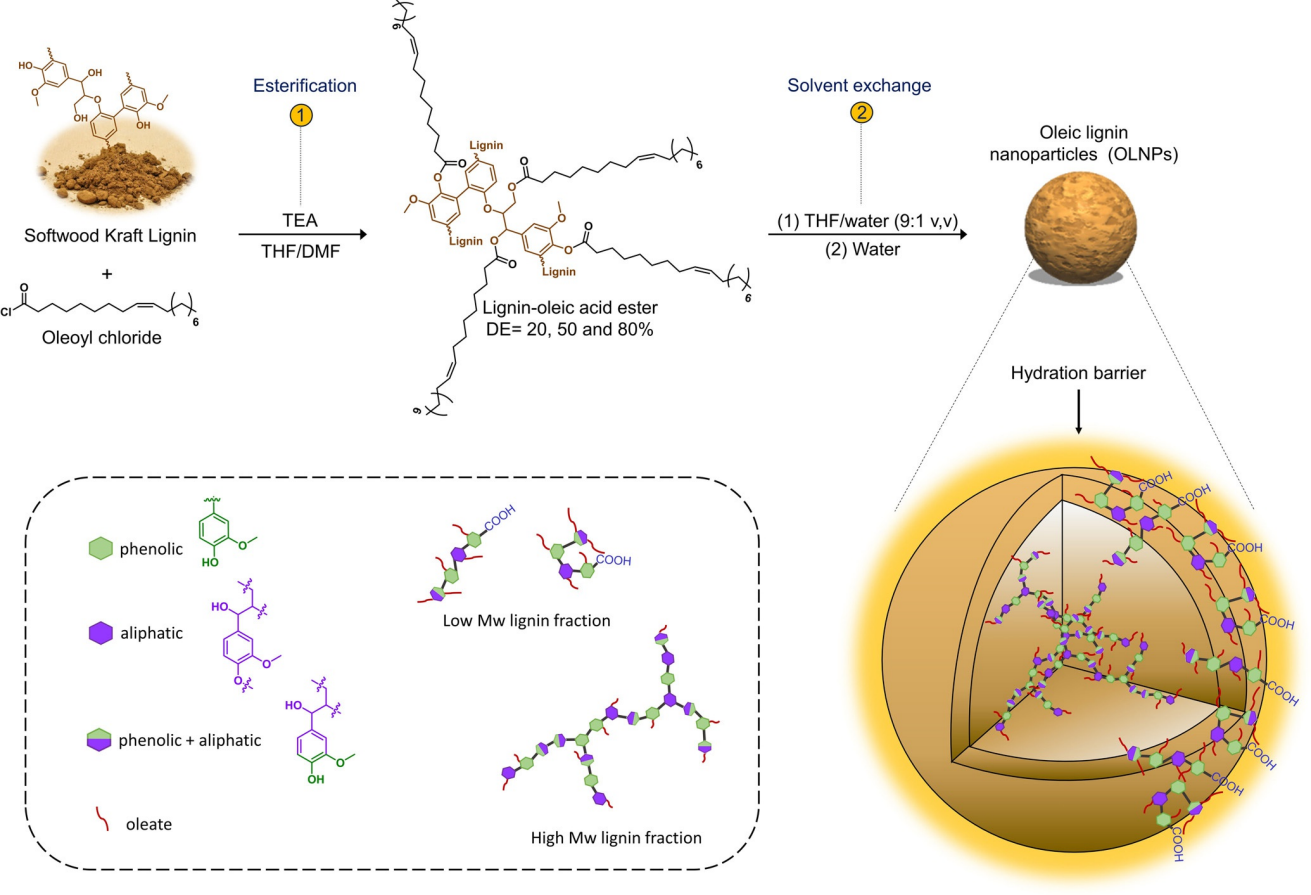
Illustration of the preparation of oleic lignin nanoparticles (OLNPs): 1) Base‐catalyzed esterification of SKL with oleoyl chloride; 2) production of OLNPs via solvent exchange precipitation from lignin‐oleic acid esters. The orange color in the representation of OLNPs indicates the presence of a hydration barrier produced by the exposure of esterified oleate chains close to the surface of the OLNPs. The illustration in the top row of the spherical OLNP was adopted from Ref. [32].

Here, we report the synthesis of oleic lignin nanoparticles (OLNPs) with unprecedented long‐term stability under acidic (pH 2.0) and alkaline (pH 12) aqueous conditions, and the preparation of functional coatings and adsorbents. The enhanced stability of the OLNPs that have been derived from lignin‐oleic acid esters is related to the accumulation of the oleic acid chains close to the particle surfaces, which acts as a hydration barrier that retards the ionization of phenolic groups under alkaline conditions and protonation of carboxylic groups in acidic media (Figure 1). We show that the hydration barrier can be tuned by the degree of esterification (DE) and thus “program” the stability of the particles. The enhanced stability of the OLNPs enables, for the first time, covalent functionalization of non‐cross‐linked lignin particles in the dispersion state via acid‐ and base‐catalyzed ring‐opening reactions. Finally, we demonstrate as a proof of principle that functionalized OLNPs can be used to prepare functional anticorrosive coatings and pH‐switchable adsorbents for removal of organic dyes from wastewater.

## Results and Discussion

**Figure 2:**
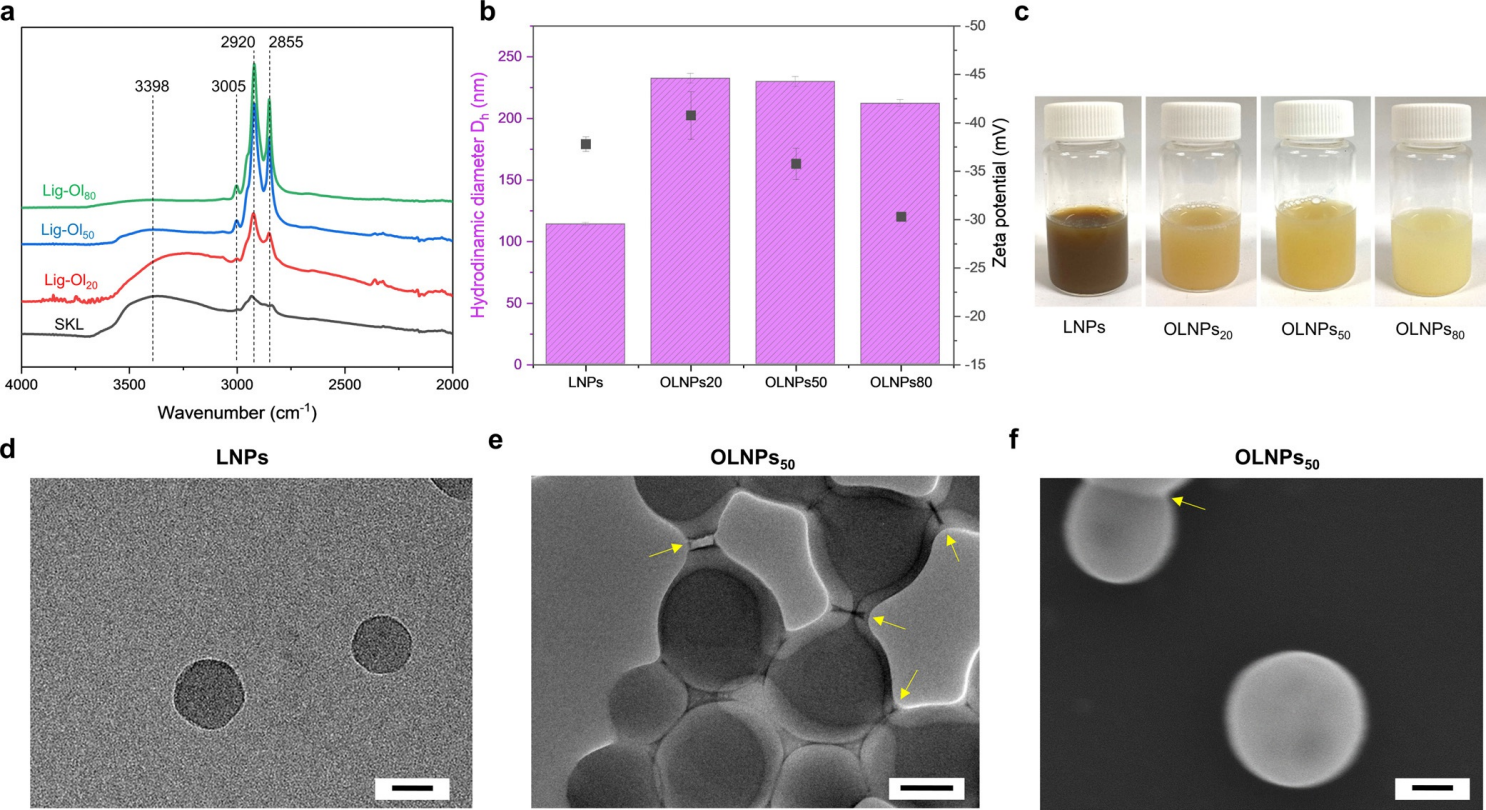
Characterization of lignin‐oleic acid esters and OLNPs: a) FTIR spectra of SKL and lignin‐oleic acid esters with different degrees of esterification (20–80 %). b) Size distribution and zeta potential of lignin particles (LNPs and OLNPs) prepared in this work. c) Digital images of LNPs and OLNPs colloidal dispersions. Transmission electron microscopy (TEM) images of d) LNPs and (e) OLNPs_50_. f) Scanning electron microscopy (SEM) image of OLNPs_50_. Scale bars (100 nm). The error bars in b represent +/‐ standard deviation (SD) from the mean values (*n*=3).

Our approach to solvent‐stable particles starts with the preparation of lignin‐oleic acid esters via base‐catalyzed esterification of softwood Kraft lignin (SKL) with oleoyl chloride (Figure 1). The degree of esterification (DE) was varied using 20, 60, and 130 % molar ratios of oleoyl chloride relative to the initial amount of hydroxyl groups present in SKL. In total, three samples with experimental DE of 18 %, 52 %, and 81 % (Lig‐Ol_20_, Lig‐Ol_50_, and Lig‐Ol_80_) were obtained as determined by ^31^P NMR spectroscopy (Supporting Information, Figure S1 and Table S1). The successful esterification process was confirmed by FTIR through the clear and progressive decrease of the hydroxyl stretching band (O‐H, 3400 cm^−1^) and the incremental appearance of the stretching bands corresponding to the double bond (=C−H, 3000 cm^−1^) and alkyl chain (2920–2840 cm^−1^) respectively, as the molar ratio of oleoyl chloride to lignin increased (Figure 2 a). Quantitative ^31^P NMR spectroscopy data also indicated a higher selectivity towards the esterification of aliphatic hydroxyl groups compared to their phenolic counterparts owing to a combination of steric and thermodynamic effects, as reported previously (Figure S2).^[45]^ Here, it is important to note that, albeit using a 130 % molar ratio, the maximum degree of functionalization that could be achieved corresponded to a DE of around 80 %. This fact originates from the higher reactivity of the accessible aliphatic and phenolic hydroxyl groups compared to the moieties with steric hindrance to the bulky oleoyl chloride. After the esterification process, complete removal of free oleic acid was not possible, especially in the case of Lig‐Ol_80_ because the quantity of oleoyl chloride in the reaction was above the stoichiometric level. However, semi‐quantification of free oleic acid from the ^31^P NMR spectra showed less than 10 wt‐% for Lig‐Ol_80_ and 5 wt‐% and 1 wt‐% for Lig‐Ol_50_ and Lig‐Ol_20_, respectively (Table S1 and Figure S3). Owing to these low concentrations of free oleic acid and envisioning that in the acid form it would be encapsulated in the inner part of the particles, no further purification process was applied. In fact, as will be shown later in this manuscript, the free oleic acid did not have a significant contribution to the formation and structure of the particles.

The preparation of OLNPs from the lignin‐oleic acid esters (Lig‐Ol_20‐80_) was performed via a solvent‐exchange methodology adapted from previous work (Figure 1).^[46]^ A binary solvent, tetrahydrofuran‐water at a mass ratio of 9:1 was selected to ensure complete dissolution of the starting materials, and particles were formed by the progressive addition of water. For comparative purposes, regular LNPs were also prepared. In all cases, stable OLNPs could be obtained regardless of the DE (Figure 2 b and Table S2). The results of dynamic light scattering (DLS) revealed no significant differences in particle sizes among the three OLNPs (Figure 2 b). Direct comparison between LNPs and OLNPs points out a significant difference in particle sizes (Figure 2 b) that suggests the presence of hydrophobic oleic fatty acid chains in the inner part of the particle during the precipitation/self‐assembly process, which leads to an increase in the size of the particles. As mentioned before, the particle size was unaffected by the increasing DE, which translates to an increased concentration of hydrophobic fatty acids chains within the particles. The formation of LNPs is governed by the molecular size and (in)solubility of the lignin molecules in such a way that the stable particles have relatively more hydrophobic cores composed of higher molecular weight lignin molecules, and hydrophilic surfaces consisting of relatively smaller lignin molecules.^[22]^ The esterification reaction will increase the hydrophobicity of the modified lignin and also change its structure. We know that SKL is a heterogeneous and polydisperse material composed of branched amphiphilic molecules.^[47]^ Carboxylic acid and phenolic hydroxyl groups are enriched in the low molecular weight fraction that constitutes the charged shell of OLNPs.[^16^, ^48^] Building on these facts, a hypothesis can be set forth that the amphiphilic lignin oleate molecules orient so that their carboxylic groups are exposed to the water phase surrounding the particles. As a result, the oleate chains are forced to collapse and associate at the surface by the hydrophobic effect, minimizing their contact with water (Figure 1). Consequently, the OLNPs exhibit charged surfaces with oleate chains acting as efficient hydration barrier that prevents permeation of water into the particles.

The transmission electron microscopy (TEM) micrographs of regular LNPs and OLNPs_50_ (Figure 2 d and e) show that all the particles are spherical and that the OLNPs_50_ appear to have core–shell structures with sticky shells and a tendency to agglomerate upon drying when the electrostatic repulsion forces are no longer effective (Figure 2 e, yellow arrows). The agglomeration behavior indirectly supports our hypothesis that the oleate chains rest on the particle surfaces. Scanning electron microscopy (SEM) images also confirm the formation of spherical and uniformly shaped OLNPs within the investigated DE range (Figure 2 f and Figure S4). Digital images of colloidal dispersions also reveal a decrease in the brown color intensity as the DE increases (Figure 2 c). This behavior arises from reduced π‐π interactions between the aromatic rings of lignin due to conformational changes caused by the introduction of oleic fatty acid chains, as evidenced by the non‐linear decrease in the UV absorbance (Figure S5), and previously shown by DFT calculations.^[49]^ This visual color change could also be of interest for applications such as sunscreens and paints, where the brown color of regular LNPs hampers production of formulations with color intensities matching consumer preferences that also differ based on the geographical regions.[^50^, ^51^, ^52^]

**Figure 3:**
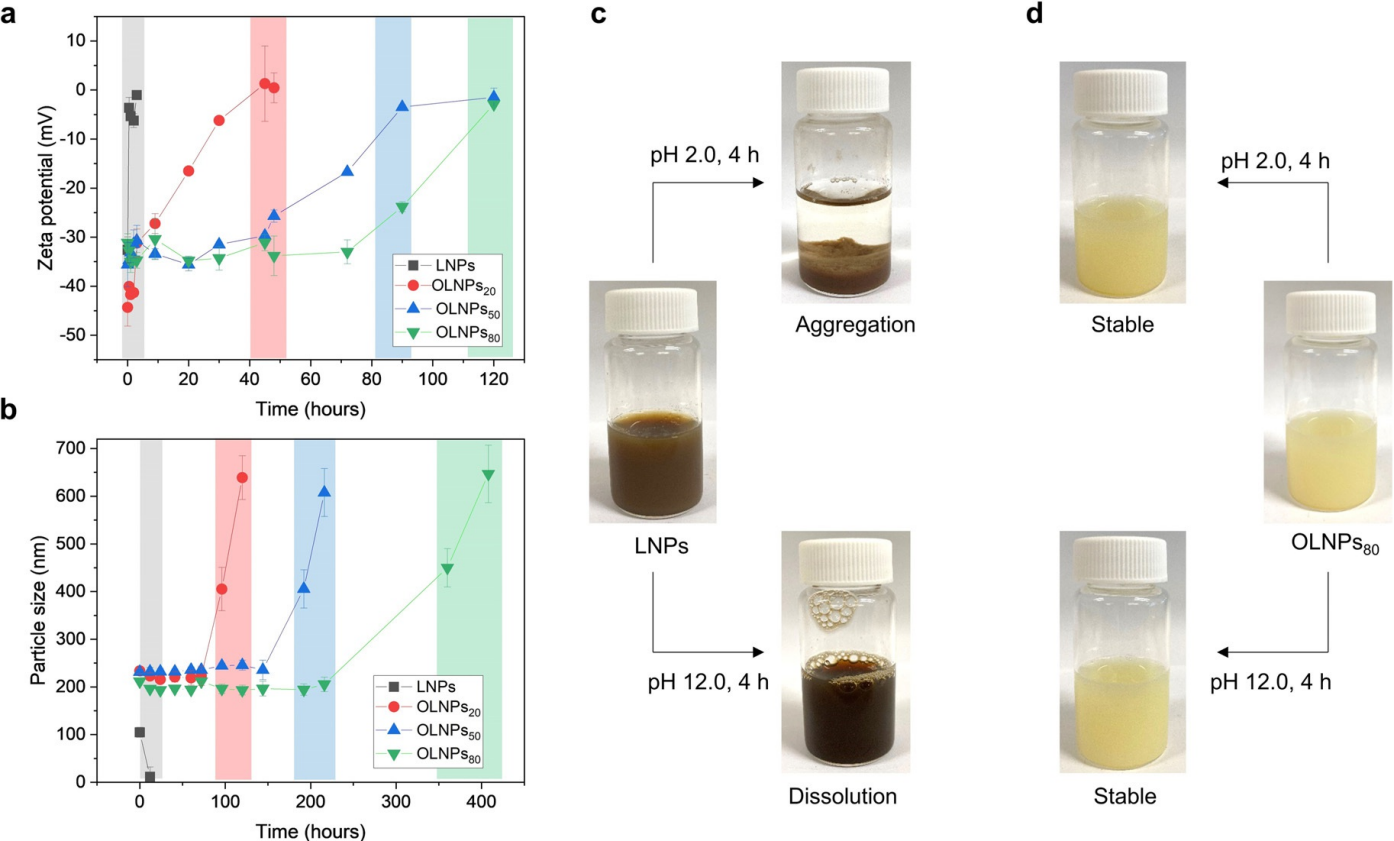
Stability of LNPs and OLNPs in different pH media: a) Evolution of zeta potential for LNPs and OLNPs at pH 2.0. b) Evolution of particle size for LNPs and OLNPs at pH 12.0. The colored dashed sections in (a) and (b) indicate the time‐dependent aggregation/dissolution of different particles. (c), and (d) digital images of the evolution of LNPs and OLNPs_80_ colloidal dispersions after exposure at different pH values (2.0 and 12.0) for 4 hours. The error bars in *a* and *b* represent +/‐ standard deviation (SD) from the mean values (*n*=3). Note that regular LNPs employed for stability assays were produced using an acetone‐solvent as the initial mixture.

With the OLNPs available as colloidal aqueous dispersions, the original idea was focused on producing internally stabilized particles via the radical cross‐linking of the double bond situated in the inner core–shell of OLNPs. However, in contrast to regular LNPs, initial colloidal stability assays in acidic (pH 2.0) and basic (pH 12.0) conditions revealed an unprecedented improvement in the stability of OLNPs. While LNPs aggregate and dissolve in 4 hours under acidic and basic conditions, OLNPs_80_ remained intact as determined by visual inspection and particle size measurements (Figure 3, compare c and d and Figure S6). Encouraged by these findings, kinetic experiments under basic and acidic conditions were conducted with all OLNPs compositions (Figure 3 a and b). Time‐dependent zeta potential measurements under acidic conditions revealed that regular LNPs are rapidly charge‐neutralized and start to aggregate within 30 minutes owing to complete protonation of carboxylic acid groups (Figure 3 a, black squares, and Figure S7), while the reduction of the negative surface charge of OLNPs is much slower and decreases with increasing DE. For instance, OLNPs_20_ was charge‐neutralized after 48 hours, while OLNPs_80_ remained anionic for more than 100 hours (Figure 3 a and Figure S8). Particle size kinetic experiments under basic conditions also revealed an identical stability dependence behavior as a function of DE (Figure 3 b). In this case, complete dissolution of regular LNPs occurs after 12 hours (Figure 3 b, black squares and Figure S9), while aggregation of OLNPs_20_ took place only after 5 days and OLNPs_80_ remained stable for more than 15 days (Figure 3 b, compare red circles with green inverse triangles and Figure S10). The evolution of polydispersity (PDI) values for all particle compositions was also evaluated, and a similar trend as that for the particle size was observed with an increase in PDI values over time due to the aggregation and precipitation of the particles (Figure S11). Since the destabilization of OLNPs was associated with an increase in particle size under basic conditions, we postulate that the underlying reason was dissolution of the charged lignin molecules and loss of electrostatic repulsion between the particles. In summary, we suggest that the main reason behind the extraordinary stability of OLNPs stems from the hydration barrier originating from the oleate chains associated with particle surfaces by hydrophobic interactions.

The unequivocally higher stability of OLNPs in acidic and basic media in contrast to regular LNPs envisions the possibility of programming their stability on‐demand by tuning the DE, which would be useful for active loading and release of pesticides for advanced plant protection.^[53]^ We attribute this significant increase in the stability of OLNPs to the hydration barrier caused by the presence of oleate chains resting on the surfaces of OLNPs. Under acidic and basic conditions, these hydrophobic chains would act as an external hydrophobic membrane, hindering access and thus protonation of carboxylic acid groups and ionization of phenolic hydroxyl groups. The stability dependence as a function of DE that was observed in both cases (Figure 3 a and b), together with the evidence of a core–shell structure also observed by TEM (Figure 2 e) supports this hypothesis, revealing OLNPs_80_ as the most stable colloidal system due to the major presence of these hydrophobic chains. Here, it is important to highlight that enhanced stability under basic conditions is also associated with a lower concentration of available ionizable hydroxyl groups due to the esterification process.

**Figure 4:**
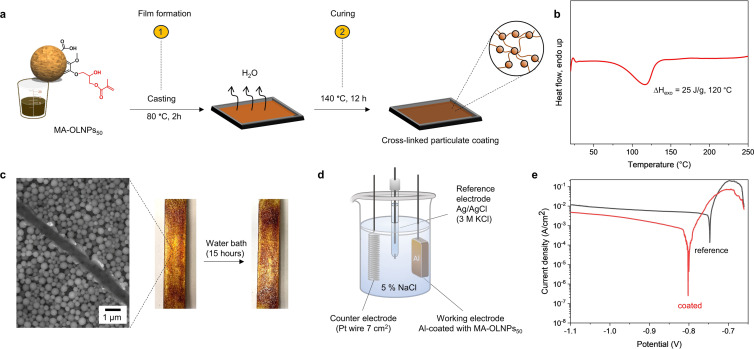
Application of MA‐OLNPs_50_ as an anticorrosion coating for metal surfaces: a) Illustration of the preparation of MA‐OLNPs_50_ ‐coated Al specimens: 1) Solution deposition of MA‐OLNPs_50_ and evaporation at moderate temperature; 2) curing of MA‐OLNPs_50_ in the presence of AIBN at elevated temperature and prolonged time. The illustration of the spherical OLNP was adopted from Ref. [32]. b) DSC thermogram corresponds to the dynamic curing at 10 °C min^−1^ of freeze‐dried MA‐OLNPs_50_ in the presence of AIBN. c) SEM image of a diagonally scratched surface of MA‐OLNPs_50_ ‐coated Al specimen, and digital images before and after the exposure of cured MA‐OLNPs_50_ ‐coated Al specimen to saline water (5 % NaCl) during 15 hours. d) Experimental setup for electrochemical measurements. e) Potentiodynamic polarization curves (Tafel plots) for coated (MA‐OLNPs_50_ ‐coated Al, red line) and non‐coated aluminum substrates (reference, black line) after exposure to a 5 % NaCl solution at 25 °C for 15 hours.

Having demonstrated the stability of the particles in both extreme conditions, we also explored the possibility of conducting covalent surface functionalization in the same media, via acid‐ and base‐catalyzed ring‐opening reactions using glycidyl methacrylate (GMA) and (glycidyl trimethylammonium chloride (GTMA), as oxirane electrophilic sources (Figure S12). OLNPs_50_ were selected as the model particles due to their moderate stability and availability of hydroxyl groups. First, successful functionalization under acidic conditions was proved by the preparation of methacrylate‐OLNPs_50_ (MA‐OLNPs_50_) after the reaction of OLNPs_50_ with GMA (Figure S12a, right). ^1^H NMR spectroscopy of freeze‐dried MA‐OLNPs_50_ demonstrated successful functionalization as evidenced by the appearance of signals corresponding to the methacrylate system (5–6.6 ppm; Figure S12c). DSC measurements were also conducted to prove the curing behavior of MA‐OLNPs_50_ in the presence of a thermal radical initiator. The observation of a single exothermic enthalpy peak (Δ*H*_exo_) without any shoulder or tail demonstrated that homopolymerization of the methacrylate moiety took place, envisioning the possibility of using MA‐OLNPs_50_ as curing agents (see below; Figure 4 b).^[54]^ In the case of base‐catalyzed functionalization, the preparation of cationized particles (*c*‐OLNPs_50_) with pH‐switchable surface charge (Figure S12a, left) also proved an efficient covalent surface modification. *c*‐OLNPs_50_ displayed a cationic net charge below pH 4.0, and an anionic net charge above pH 7.0 (Figure S12b), without a significant difference in particle size in comparison to original particles regardless of their net charge (Figure S13). Between pH 4.0 and 7.0, the particles underwent a transition from positive to negative charge (Figure S12b, red dashed section), undergoing reversible aggregation as a consequence of protonation‐deprotonation of carboxylic acid groups (p*K*_a_=4). Similar pH‐responsive charge behavior was reported earlier by Sipponen et al. for non‐covalent functionalized LNPs with water‐soluble cationic lignin,^[34]^ and Zou et al. for covalent functionalized LNPs with GTMA.^[44]^ However, the particles presented herein have the advantages of pH stability, ion exchange resistance, and no need for an internally cross‐link process to conduct the functionalization. Here, it is important to note that both functionalization processes constitute the first examples of surface covalent functionalization of LNPs without the need for cross‐linkers for stabilization. Aside from cationization or acrylation reactions, we also hold a view that the high versatility of our particles would open new possibilities for additional surface covalent reactions, as is the case of the Mannich reaction or specific applications such as coatings,^[55]^ where the particles can resist leaching under harsh environmental conditions. Last but not least, albeit the preparation of OLNPs has been previously reported,^[56]^ stability in extreme pH conditions and covalent surface functionalization have remained unexplored, limiting their potential applications.

To confirm the potential application of functionalized OLNPs_50_, two applications were demonstrated: 1) MA‐OLNPs_50_ as an anticorrosion coating for metal surfaces, and 2) *c*‐OLNPs_50_ as selective adsorbent nanomaterials for dye removal from aqueous solutions. First, the inherent ability of lignin to act as a redox‐active radical scavenger, combined with the ability to thermally cross‐link the particles, prompted us to investigate their use as an anticorrosion coating for metal surfaces. Aluminum (Al) was selected as a model metal substrate since it is the most consumed nonferrous metal, and can be easily corroded in non‐neutral pH and chlorinated solutions.[^57^, ^58^] MA‐OLNPs_50_ ‐coated Al specimens were obtained by deposition of MA‐OLNPs_50_ dispersion followed by evaporation and thermal curing process (Figure 4 a).

The stability of the coating, which is critical for the final application, was investigated in saline water. Figure 4 c depicts MA‐OLNPs_50_ ‐coated Al specimens before and after immersion in saline solution for 15 hours. No significant changes were observed, confirming the excellent stability of the coating without leaching or disintegration of MA‐OLNPs_50_. In contrast, the MA‐OLNPs_50_ ‐coated Al specimen produced under identical conditions but without the curing step disintegrated in the course of the same process, pointing out the importance of the functionalization of the particles to reach curable and stable coatings (Figure S14). Additionally, MA‐OLNPs_50_ ‐coated Al were also found to be stable under different harsh environments such as acidic (pH 2.0) and basic (pH 12.0), which would be of great interest to alternative manufacturing processes. SEM images of MA‐OLNPs_50_ ‐coated Al specimen also revealed the presence of MA‐OLNPs_50_ efficiently covering the Al surface without losing their spherical morphology after curing (Figure 4 c). This morphological rigidity was achieved despite the fact that the melting point of Lig‐Ol_50_ (precursor of MA‐OLNPs_50_) was 74 °C in contrast to a glass transition temperature of 156 °C and a lack of melting point in the pristine Kraft lignin (Figure S15), as previously reported.^[45]^ OLNPs_50_ maintain their spherical morphology most probably owing to the crosslinking of the particle surfaces that contain acrylate moieties, thereby limiting the melting process to the inner part of the particles. Last but not least, potentiodynamic polarization studies were also carried out to evaluate MA‐OLNPs_50_ as an anticorrosion coating (Figure 4 d and e). Figure 4 e depicts the potentiodynamic polarization curves for non‐coated (bare Al) and coated (MA‐OLNPs_50_ ‐coated Al) specimens after 15 hours of immersion in a corrosive media of 5 % NaCl aqueous solution. The corrosion resistance was measured through the corrosion current density (CCD) corresponding to the intersection point between the anodic and the cathodic polarization curves (Figure 4 e). The lower the CCD value, in general, the better the anti‐corrosion behavior of the material, in this case, Al. After exposure to saline solution, the bare Al specimen showed three orders of magnitude higher CCD value (1.35×10^−4^ A cm^−2^) than the MA‐OLNPs_50_ ‐coated Al specimen (2.54×10^−7^ A cm^−2^). This fact, together with only a small shift in the corrosion potential with respect to bare Al sample (−0.8 V and −0.74 V vs. Ag/AgCl for MA‐OLNPs_50_ ‐coated Al and bare Al, respectively) indicates unequivocally a good barrier behavior and an effective protection against corrosion owing to the MA‐OLNPs_50_ coating. It is worth noting that our findings compare favorably to previous reports on the performance of lignin‐based and other anticorrosion coatings for aluminum (Table S3).[^59^, ^60^, ^61^] Moreover, our approach avoids the use of toxic chemical reagents such as isocyanates or epoxy compounds as cross‐linking agents, which could hinder their transfer to technological applications.

**Figure 5:**
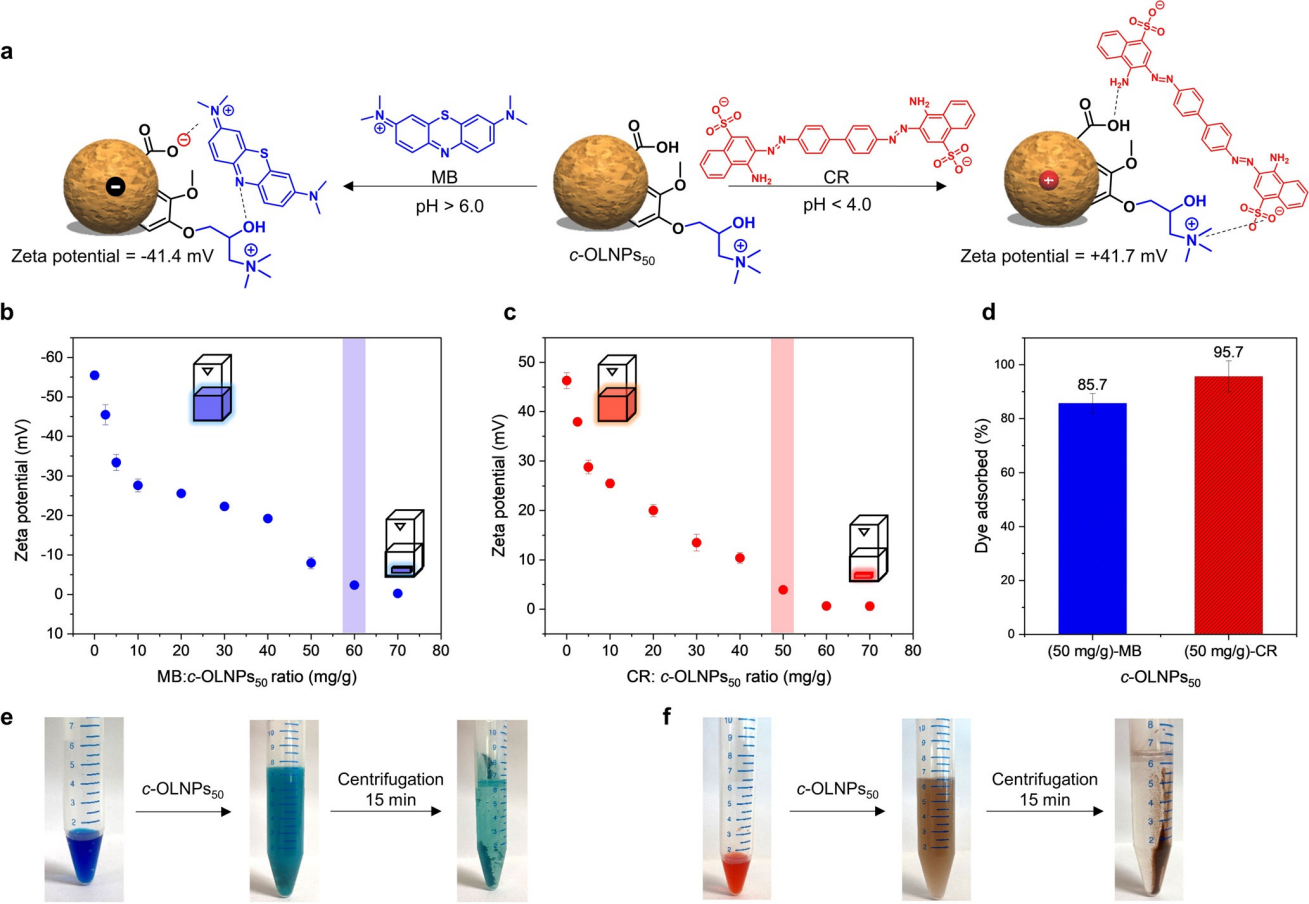
Application of *c*‐OLNPs_50_ in dye adsorption in aqueous solutions: a) Illustration of non‐covalent electrostatic interaction between *c*‐OLNPs_50_ with positive‐ (methylene blue, MB) and negative‐charged (Congo red, CR) dyes as a function of pH. The illustration of the spherical OLNP was adopted from Ref. [32]. b) Evolution of zeta potential after the addition of MB at pH 7.0. c) Evolution of zeta potential after the addition of CR at pH 3.5. Measurements were conducted after holding the mixture for 10 min at room temperature. Measurements at longer times (2 h) did not show any difference. d) Dye adsorption removal capacity of *c*‐OLNPs_50_ towards MB and CR. e),f) Digital images of dye removal from aqueous solutions using *c*‐OLNPs_50_. Error bars in (b), (c), and (d) represent one standard deviation (SD) from the mean values (*n=*3).

In the case of *c*‐OLNPs_50_, the pH‐switchable surface charge of *c*‐OLNPs_50_ was exploited to adsorb methylene blue (MB) and Congo red (CR) as cationic and anionic model compounds, respectively, from aqueous solutions via non‐covalent electrostatic interactions (Figure 5 a). Dosing MB at increasing dry weight ratios relative to *c*‐OLNPs_50_ (negatively charged at neutral pH) revealed that 60 mg g^−1^ is required for complete neutralization of the surface charge (Figure 5 b and Figure S16). In the case of CR, almost identical results were obtained when the particles were positively charged under acidic conditions (Figure 5 c). This marks unequivocally the optimal concentration at which both systems showed an excellent ability to remove the corresponding dyes from aqueous solutions in a short time (10 minutes) (Figure 5 d, e, and f). Reusability of the particles was also evaluated by neutralization of particle charge at the isoelectric point (pH 4.5, Figure S12b), and re‐dispersion of the dye. However, only moderate yields of the particles could be recovered (40 %) due to the strong interfacial interactions between the particles and dye molecules. In addition to the long‐range electrostatic attractive forces driving the adsorption processes, short‐range stabilization by Van der Waals forces, hydrogen bonding, and π–π stacking between aromatic groups also take place in both cases, contributing to the enhanced stability of the dye‐particle complexes. This high affinity towards both target dyes, together with the versatility to operate as net positive or negative nanoparticles, by simple pH regulation, demonstrates the potential of *c*‐OLNPs_50_ as a universal adsorbent material, especially for water purification and recovery of active substances,^[62]^ where the high surface area of the nanoparticles and rapid adsorption are crucial to securing an efficient process.^[63]^ In spite of their relatively lower adsorption capacity (mg g^−1^) compared to other lignin‐based adsorbents, *c*‐OLNPs_50_ exhibit the most efficient percentage removal of dyes from aqueous solutions over time (Table S4). In this context, it is also important to mention that esterification of lignin with fatty acids is already implemented in industry,^[64]^ alternative esterification routes have been reported,[^65^, ^66^] and the production of LNPs via solvent exchange methodology has been assessed to be an industrially scalable process.^[67]^ Taken together, these facts suggest that OLNPs may find industrial applications, as stated before.

## Conclusion

We have reported the preparation of lignin oleate nanoparticles (OLNPs) that are stable in acidic and basic pH for several days. This unprecedented stability was independent of any covalent cross‐linking and enables using robust acid‐ and base‐catalyzed reactions for surface‐modification of the particles in aqueous dispersions. The versatility of the obtained particles was proven by two different surface functionalization processes in acidic and alkaline media. The resulting particles were demonstrated as water‐resistant anti‐corrosive coatings and pH‐switchable adsorbents in water purification. In both cases, the superior stability of the nanoparticles translates into competitive end‐use performance. Finally, we anticipate that the straightforward synthetic methodology will open new avenues for the application of lignin‐based particles in advanced materials. For example, in addition to the methacrylation and cationization demonstrated in this work, a broad range of chemistries is now accessible for their surface functionalization for new applications.

## Conflict of interest

The authors declare no conflict of interest.

## Supporting information

As a service to our authors and readers, this journal provides supporting information supplied by the authors. Such materials are peer reviewed and may be re‐organized for online delivery, but are not copy‐edited or typeset. Technical support issues arising from supporting information (other than missing files) should be addressed to the authors.

Supporting Information
