# Supplementary material for: Unravelling the Hydration Barrier of Lignin Oleate Nanoparticles for Acid‐ and Base‐Catalyzed Functionalization in Dispersion State
Source: Angew Chem Int Ed Engl. 2021 Aug 3;60(38):20897–905. doi: 10.1002/anie.202106743 (PMC8518943; doi:10.1002/anie.202106743)
Supplement: Supplementary file 1 — Supporting Information [file ANIE-60-20897-s001.pdf]

Angewandte Chemie
Eine Zeitschrift der Gesellschaft Deutscher Chemiker

Supporting Information

Unravelling the Hydration Barrier of Lignin Oleate Nanoparticles for Acid- and Base-Catalyzed Functionalization in Dispersion State

Adrian Moreno, Jinrong Liu, Robin Gueret, Seyed Ehsan Hadi, Lennart Bergström, Adam Slabon, and Mika H. Sipponen**

anie_202106743_sm_miscellaneous_information.pdf

Materials

All lignin materials prepared in this work (LNPs, lignin-oleic acid esters, and OLNPs) were prepared from BIOPIVA™ 100 pine Kraft lignin (SKL) (UPM, Finland), previously characterized.[1] All the chemicals and solvents were purchased from Sigma-Aldrich, Fischer, and VWR and were used as received unless noted. Glycidyl methacrylate (GMA) was passed through a short basic alumina column to remove the inhibitor before the functionalization of $OLNPs_{50}$. Azobisisobutyronitrile (AIBN) was recrystallized in methanol before the curing reactions. As metallic substrates, aluminum 6061 alloys with a rectangular shape and dimensions of 0.5 x3 cm using a gauge length of 1 cm were used. Deionized (DI) water was used throughout the experiments. Dialysis was performed on Spectra/Por® tubes with an MWCO of 6–8 kDa against deionized water.

Methods

NMR spectroscopy. ^1H NMR spectra were recorded on a Bruker DRX 4OO NMR instrument at 25 °C in $CDCl_3$ containing tetramethylsilane as an internal standard. ^{31}P NMR spectra were recorded following a previously described methodology.[2] Briefly, a 90° pulse angle, inversed gated proton decoupling, and a delay time of 10 s. were used. For the analysis, 256 scans with a 6 s delay and a total runtime of 30 minutes were used for each sample. Three replicated experiments were conducted, and the mean value of one standard deviation is reported. **Transition Electron Microscopy (TEM)** images were recorded on a JEM-2100F operating with an accelerating voltage of 200 kV. Colloidal dispersions of LNPs and OLNPs were previously diluted by a factor of 1:40, followed by the deposition and evaporation onto a carbon-coated copper grid. Gatan Inc. software was used to process the images. **Scanning Electron Microscopy (SEM)** images were recorded on a JEOL JSM-7401F (JEOL Ltd., Japan) operating at 2−5 kV. Colloidal dispersions of OLNPs were previously diluted by a factor of 1:40, followed by the deposition and evaporation of one droplet into a silicon wafer matrix for the SEM investigation. **Differential Scanning Calorimetry (DSC)** measurements were performed on Netzsch DSC 214 Polyma with N_2 as the purge gas (50 mL/min) and using a heating rate of 10 °C/min in the 25–250 °C temperature range. Calibration was performed using an indium standard for heat flow calibration and a zinc standard for temperature calibration. **Dynamic Light Scattering (DLS)** measurements were performed on a Zetasizer Nano ZS (Malvern, UK). The zeta potential was determined using a dip cell probe. LNPs and OLNPs were diluted by a factor of 30 with deionized water respectively before the analysis. Zeta potential measurements in the presence of 9 mM phosphate buffer were also conducted to elucidate the effect of the electrolyte in

the solution (Figure S17). **Corrosion test.** Potentiodynamic polarization tests were performed on bare Al and Al-coated MA-OLNPs$_{50}$ specimens. These electrochemical experiments were performed using a Biologic SP150 potentiostat and a three-electrodes glass cell, which consists of the coated/non-coated aluminum specimen as working electrode, an Ag/AgCl (3M KCl) as the reference electrode, and a platinum wire (7 cm^2) as the counter electrode. Potentiodynamic polarization curves were obtained using the EC-Lab software package by scanning the electrode potential at a scan rate of 0.5 mV/s in a NaCl 5% electrolyte at 25 °C. **ATR-FTIR** data were collected using a Varian 610-IR FT-IR spectrometer. The (IR) absorbance of samples was measured using an attenuated total reflection – Fourier transform infrared spectroscopic (ATR-FTIR) in the range of 450–4000 cm^{-1}.

Experimental procedures

Preparation of lignin-oleic acid esters (Lig-Ol). This procedure is representative of all the lignin-oleic acid esters produced herein. The preparation of Lig-Ol$_{80}$ is described as a representative example: 5.0 g of lignin (containing 5.94 mmol/g of total phenolic and aliphatic hydroxyl groups measured by quantitative ^{31}P NMR analysis) was dissolved for 30 min in a binary mixture of anhydrous tetrahydrofuran/dimethylformamide (30 mL/7.5 mL, v, v) containing 3.8 mL of anhydrous pyridine at 40 °C under nitrogen atmosphere. After that, 13.9 mL of oleoyl chloride (1.3 equiv vs total aliphatic and phenolic hydroxyl groups) were added with a syringe within 10 min. The reaction mixture was purged with nitrogen and kept for 15 hours at 45 °C. After that, the reaction mixture was concentrated, dissolved in dichloromethane (150 mL) and washed with brine (50 mL) 3 times. The organic fractions were collected, dried with $MgSO_4$ and concentrated under vacuum to afford Lig-Ol$_{80}$ as a dark viscous liquid with a 78 % yield. Note that Lig-Ol$_{20}$ and Lig-Ol$_{50}$ were obtained as dark powders. The degree of esterification (DE) was calculated by direct comparison of the total amount of hydroxyl groups determined by ^{31}P NMR spectroscopy between the esterified samples and SKL.

Preparation of lignin nanoparticles (LNPs and OLNPs). All lignin nanoparticles used in this work were prepared by solvent exchange methodology adapted from previous work.[3] Briefly, the synthesis included the dissolution of lignin-oleic acid esters in THF/water mixture (mass ratio 9:1), insoluble impurities were removed by filtration, and OLNPs produced by slowly addition of deionized water (30 min) to lignin-oleic acid ester solution under stirring. After that, dispersions were concentrated by rotary evaporation and dialyzed against water for 24h to ensure complete removal of the oleic acid excess and

THF. The final aqueous dispersion of OLNPs (1 g L^{-1}) was obtained with a lignin mass yield of 83%. $LNPs_{THF}$ were produced following the same procedure to afford a final aqueous dispersion of LNPs (1 g L^{-1}) with a lignin mass yield of 87%. In the case of $LNPs_{Acetone}$, the same procedure was applied, but using an initial mixture of acetone/water mixture (mass ratio of 3:1) to dissolve SKL. The final aqueous dispersion of $LNPs_{Acetone}$ (4 g L^{-1}) was obtained with a lignin mass yield of 86%.

Basic and acid stability of LNPs and OLNPs. LNPs and OLNPs dispersions (10 mL) were adjusted to pH 12.0 and 2.0 by the addition of 0.7 mL of NaOH (0.1 M) and HCl (0.1 M), respectively. Samples were incubated under orbital shaking at 25 °C. For kinetic experiments, samples were taken at regular intervals to monitor the evolution of particle size (basic pH) and zeta potential (acidic pH). During the kinetic experiments pH increased from pH=2.0 to pH=2.2 and decreased from pH=12.0 to pH=11.7 in the acidic and alkaline media, respectively

Covalent surface functionalization in acidic conditions. Preparation of methacrylate OLNPs (MA-OLNPs50). $OLNPs_{50}$ (10 mL) dispersion was adjusted to the pH of 1.5 by the addition of 1.4 mL of HCl (1 M). After that, 160 μL of glycidyl methacrylate (GMA) was added dropwise to the solution while stirring, and the reaction mixture was heated up to 60 °C over 16 hours. To remove HCl and unreacted GMA, MA-$OLNPs_{50}$ were purified using dialysis for 24 hours until the pH reached 6.5. The curing behavior of MA-$OLNPs_{50}$ was evaluated by DSC measurement of freeze-dried MA-$OLNPs_{50}$ dispersion containing AIBN (2 wt% respect MA-$OLNPs_{50}$).

Covalent surface functionalization in basic conditions. Preparation of cationized OLNPs (*c*-OLNPs50). Cationization of OLNPs followed a similar procedure described in the literature for the cationization of internally cross-linked LNPs.[4] Briefly, $OLNPs_{50}$ dispersion (10 mL) was adjusted to pH 12.0 by the addition of 0.7 mL of NaOH (0.1 M). After that, 80 μL of glycidyl trimethylammonium chloride (GTMA) was added dropwise to the solution while stirring, and the reaction mixture was heated up to 80 °C over 1 h. To remove NaOH and unreacted GTMA, c-OLNPs50 were purified via dialysis for 24 hours until the pH reached 6.5.

Preparation of Al-coated MA-OLNPs$_{50}$ specimens. A concentrated MA-OLNPs aqueous dispersion (0.4 wt% particle content and 2 wt% AIBN, with respect to MA-OLNPs$_{50}$) obtained from the sediment, centrifugation, and re-dispersion (7000 rpm for 30 min) was used for the coating process. Al-coated MA-OLNPs$_{50}$ specimens were obtained by a deposition of MA-OLNPs$_{50}$ dispersion followed by 2 hours of water evaporation at 80 °C, and then 12 hours of thermal curing of the particles at 140 °C.

Dye removal from aqueous solutions using *c*-OLNPs50. Methylene Blue (MB) and Congo red (CR) aqueous solutions were prepared at a concentration of 1mg/mL and adjusted to pH 7.0 and 3.5, respectively. After that, 5 mL of *c*-OLNPs$_{50}$ was adjusted according to the aforementioned pH and added to the corresponding dye solutions to reach the optimal adsorption concentration (See Fig 5b and c). After holding the samples for 10 minutes to ensure effective particle-dye interaction, centrifugation (7000 rpm for 15 minutes) was performed, followed by a UV spectrophotometer analysis of residual dye content in the supernatant. The dye removal efficiency (%) was calculated by comparing the UV-vis absorption spectra of dye solutions before and after adding and precipitating with c-OLNPs$_{50}$. The wavelength for MB and CR dye was set at 663 and 500 nm, respectively.

References for experimental section:

[1] M. H. Sipponen, M. Farooq, J. Koivisto, A. Pellis, J. Seitsonen, M. Österberg, *Nat. Commun.* **2018**, *9*, 2300.

[2] M. H. Sipponen, M. Smyth, T. Leskinen, L. S. Johansson, M. Österberg, *Green Chem.* **2017**, *19*, 5831–5840.

[3] A. Moreno, M. H. Sipponen, *Nat. Commun.* **2020**, *11*, 5599.

[4] T. Zou, M. H. Sipponen, A. Henn, M. Österberg, *ACS Nano*, **2021**, *15*, 4811-4823.

Figure S1 shows the evolution of ^{31}P NMR spectra after the esterification of SKL with different oleoyl chloride molar ratio contents. A clear decrease in the aliphatic and hydroxyl groups band can be observed as an increase in the content of oleoyl chloride.

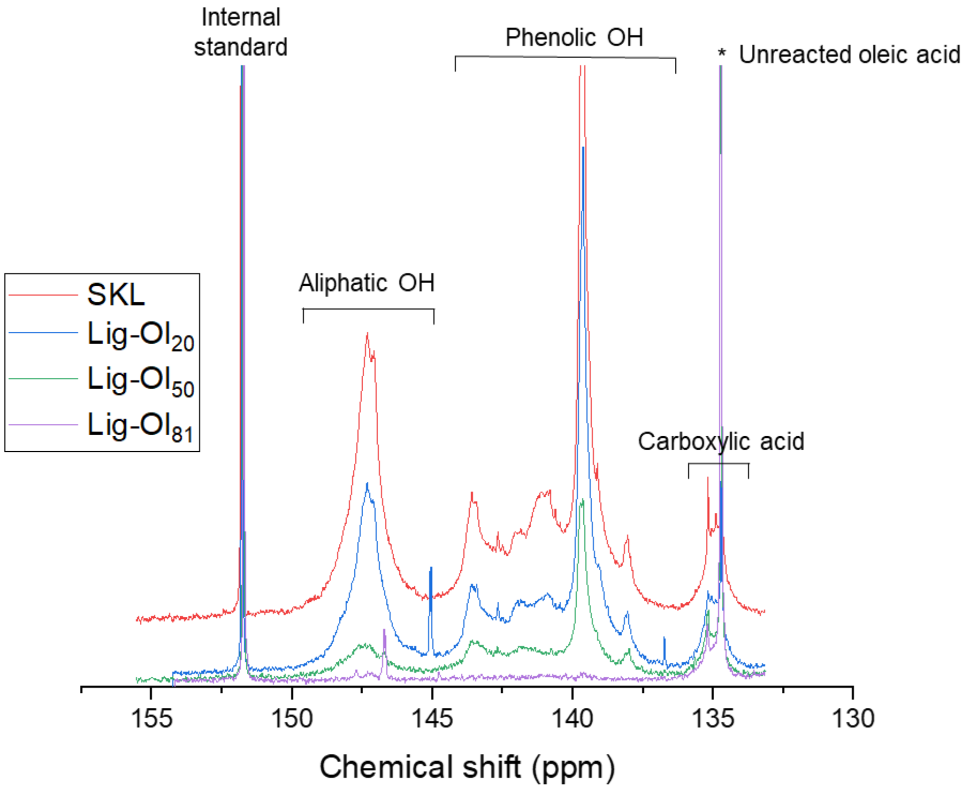

Figure S1. Quantitative ^{31}P NMR spectra of SKL and lignin-oleic acid esters at different DE.

Table S1 shows the quantitative analysis of hydroxyl groups in SKL and lignin-oleic acid esters used in this work.

Table S1. Concentration of Aliphatic and Phenolic OH of SKL and lignin-oleic acid esters according to quantitative ^{31}P NMR.

sample	aliphatic OH	phenolic OH	Total OH	wt % (free oleic acid)[b]
SWL	1.89 ± 0.04	4.05 ± 0.01	5.94 ± 0.09	-
Lig-Ol_{20}	1.34 ± 0.01	3.54 ± 0.06	4.90 ± 0.05	< 1.0
Lig-Ol_{50}	0.61 ± 0.05	2.26 ± 0.05	2.86 ± 0.01	3.5-4.5
Lig-Ol_{80}	0.24 ± 0.05	0.88 ± 0.04	1.12 ± 0.06	8.5-9.0

[a] Unit mmol/g. At least three measurements were completed for each parameter. Error ranges correspond to one standard deviation. Note, that carboxylic acid groups were excluded from the calculation due to the signal interference with oleic acid excess. [b]Semi-quantification results obtained by the deconvolution of the peak corresponding to free oleic acid and carboxylic groups of lignin (Figure S3).

Figure S2 shows the higher selectivity towards aliphatic hydroxyl groups in contrast to the phenolic counterparts as a function of the molar ration of oleoyl chloride used.

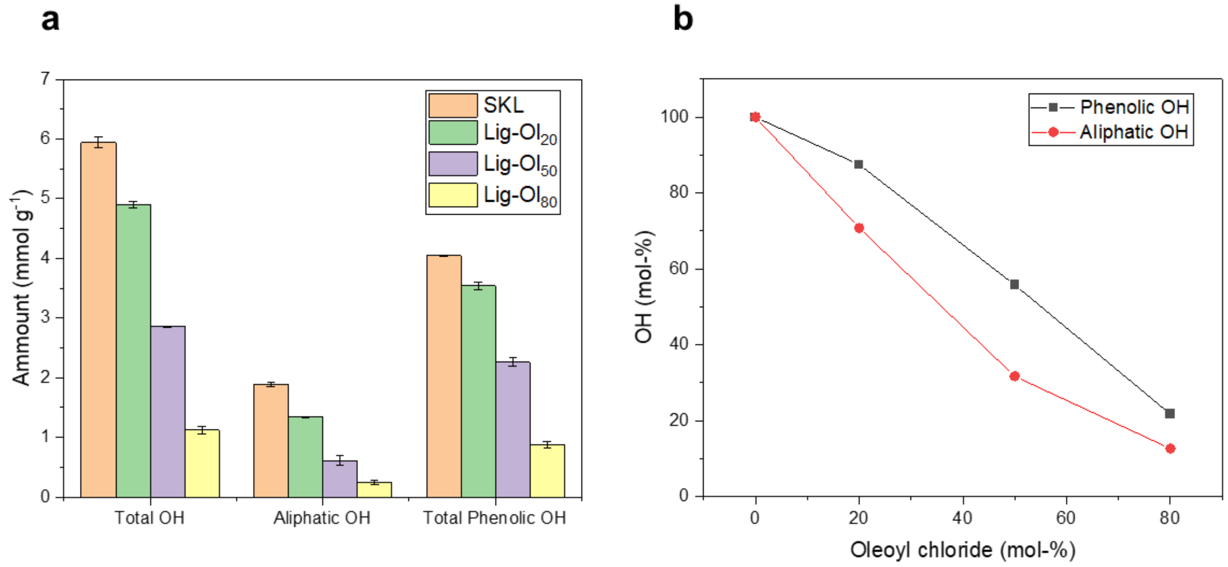

Figure S2. (a) Concentration of aliphatic and phenolic OH groups of SKL and Lig-Ol samples determined by quantitative ^{31}P NMR spectroscopy. (b) Hydroxyl group selectivity of esterified SKL with oleoyl chloride determined via quantitative ^{31}P NMR spectroscopy.

Figure S3 shows the deconvolution process of ^{31}P NMR signal corresponding to carboxylic acid in lignin and the free oleic acid for the sample Lig-Ol_{20} used to calculate the % wt of free oleic acid in the sample.

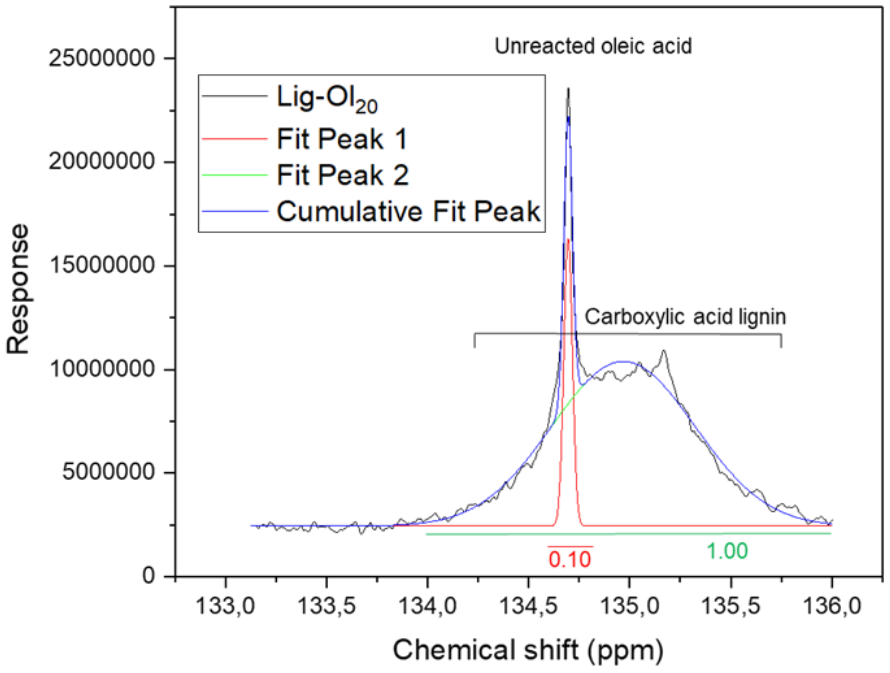

Figure S3. Deconvolution of ^{31}P NMR spectra signal for Lig-Ol_{20}

Table S2. Characteristics of lignin particles (LNPs) and oleic lignin nanoparticles (OLNPs) prepared in this work.[a] Direct comparison of zeta potential values revealed that $OLNPs_{20}$ had a slightly higher negative charge value than $OLNPs_{50-80}$, which could be attributed to minor esterification of carboxylic acid groups in the samples with higher DE.

Lignin form	hydrodynamic diameter (nm)	PDI	Zeta potential (mV)
[b]$LNPs_{Acetone}$	105 ± 1.36	0.061 ± 0.014	-32.8 ± 0.75
[c]$LNPs_{THF}$	113 ± 3.96	0.079 ± 0.017	-37.8 ± 2.75
[c]$OLNPs_{20}$	232 ± 3.96	0.097 ± 0.147	-45.7 ± 2.45
[c]$OLNPs_{50}$	229 ± 4.02	0.084 ± 0.047	-37.7 ± 1.61
[c]$OLNPs_{80}$	212 ± 2.81	0.069 ± 0.051	-35.7 ± 0.31

[a]At least three measurements were completed for each parameter. Error ranges correspond to one standard deviation. Values measured at native pH (3.8-4.2). [b]LNPs prepared via solvent exchange methodology using acetone-water as the initial solvent system for a final LNPs concentration of 4g L^{-1}. [c]LNPs and OLNPs are prepared via solvent exchange methodology using THF-water as the initial solvent system for a final LNPs and OLNPs concentration of 1g L^{-1}.

Figure S4 shows SEM characterization images of OLNPs used in this work.

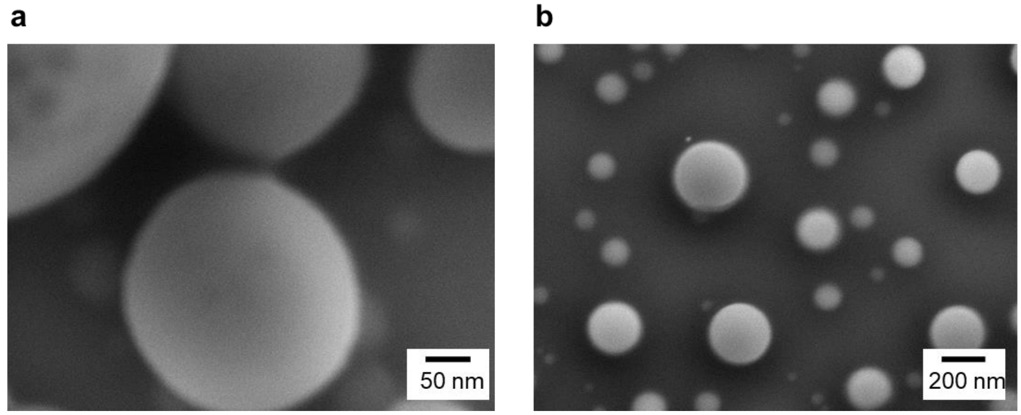

Figure S4. SEM images of (a) $OLNPs_{20}$ and (b) $OLNPs_{80}$ produced from lignin-oleic acid esters ($Lig\text{-}Ol_{20}$ and $Lig\text{-}Ol_{80}$) via solvent exchange precipitation.

Figure S5 shows the UV-Vis analysis corresponding to LNPs and OLNPs colloidal dispersions. As can be observed, there is a non-linear change in the maximum absorbance (300 nm) as a function of DE. The intensity drops dramatically from 0% (LNPs) to 20% ($OLNPs_{20}$), followed by smaller jumps as DE increases. These changes indicate a less effective absorbance of UV for OLNPs as DE increases, which has been ascribed to the reduced π–π interaction between the aromatic rings.

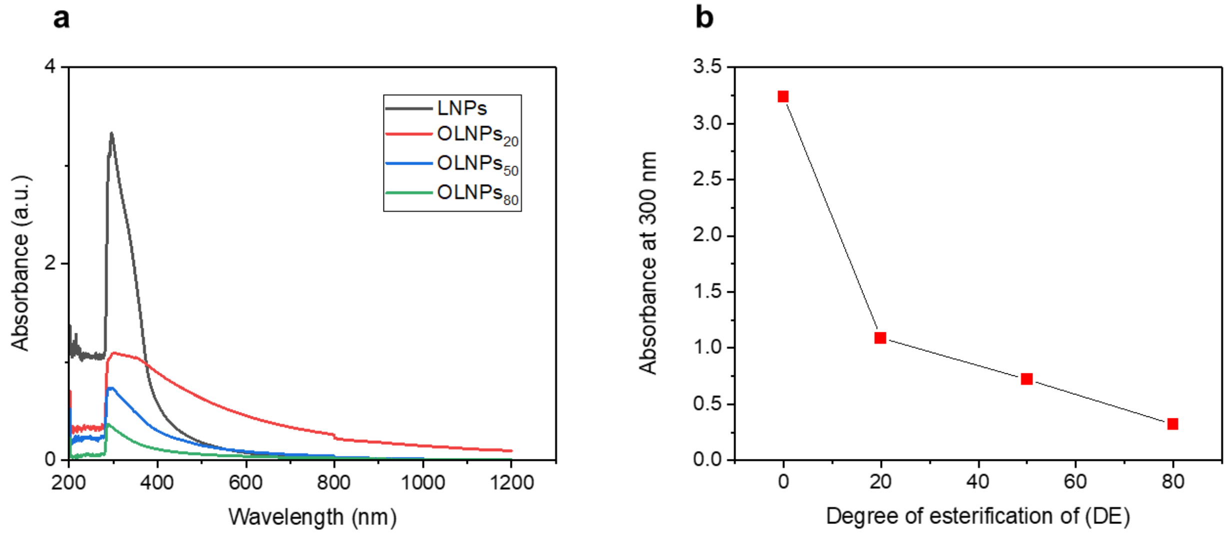

Figure S5. (a) UV-Vis absorbance of lignin nanoparticles. (b) Absorbance (at 300 nm) of LNPs and OLNPs dispersions as a function of DE.

Figure S6 shows the higher stability of OLNPs80 by insignificant changes in particle size after 4 hours of exposure to acidic (pH = 2.0) and basic (pH = 12.0) aqueous media.

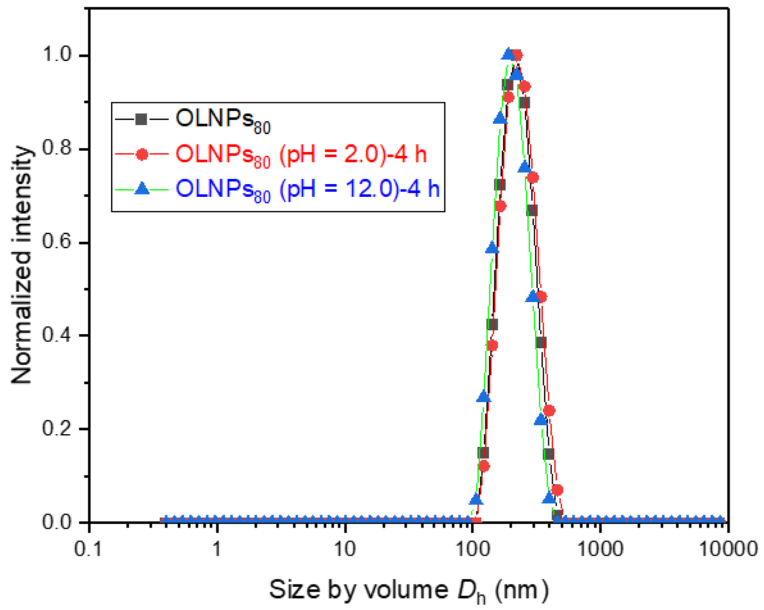

Figure S6. Evolution of particle size on OLNPs after exposure at pH 2.0 and pH 12.0 after 4 hours.

Figure S7 shows the aggregation of regular LNPs by observation of the increase in particle size and the neutralization of surface charge after exposure to acidic (pH = 2.0) for 30 min.

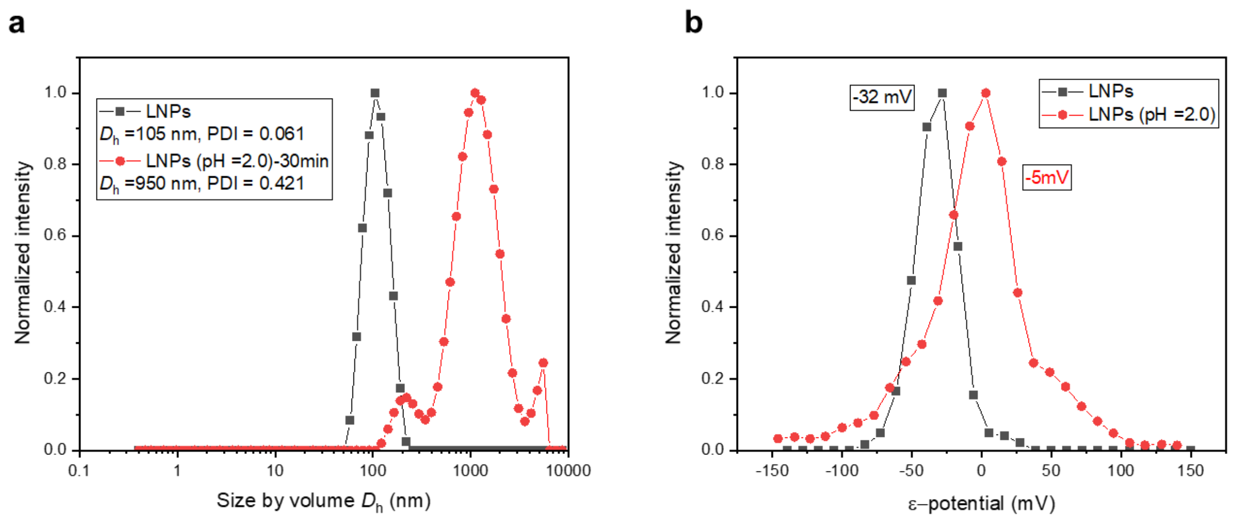

Figure S7. Evolution of (a) particle size and (b) zeta potential of LNPs after exposure at pH 2.0 for 30 min.

Figure S8 shows the stability dependence of OLNPs as a function of DE in acidic conditions. While $OLNPs_{20}$ starts to aggregate after 45 h, $OLNPs_{80}$ remains stable up to 120 hours due to a major presence of oleic fatty acid chain in the surface of the particle, which hinders protonation of carboxylic acids.

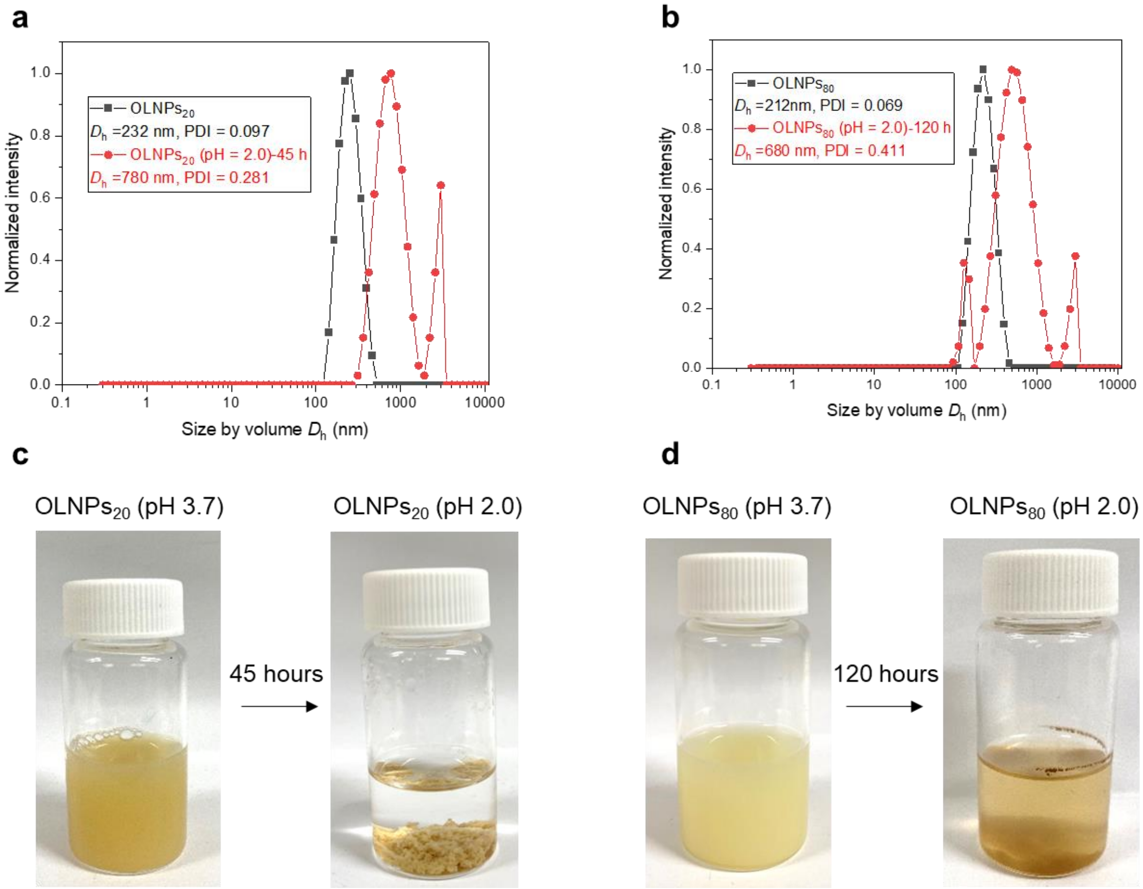

Figure S8. Evolution of particle size for (a) $OLNPs_{20}$ and (b) $OLNPs_{80}$ at pH 2.0 over time. Digital images of the (c) $OLNPs_{20}$ and (d) $OLNPs_{80}$ before and after the aggregation process.

Figure S9 shows the dissolution process of regular LNPs by observation of a decrease in the particle size and an increase in the surface charge due to ionization of phenolic groups after exposure to basic media (pH = 12.0) for 30 min.

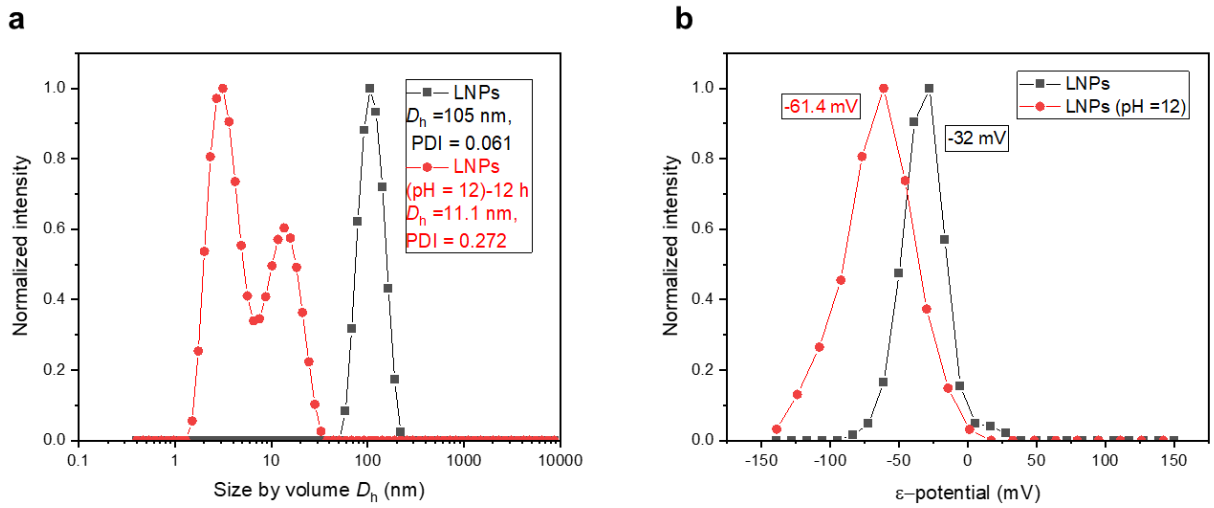

Figure S9. The evolution of (a) particle size and (b) zeta potential of LNPs after 12 hours of exposure at pH 12.0.

Figure S10 also shows the stability dependence of OLNPs as a function of DE in basic conditions. While $OLNPs_{20}$ starts to aggregate at 150 hours, $OLNPs_{80}$ remains stable up to 450 hours due to a major presence of oleic fatty acid chain on the surface of the particles hindering the ionization process, but also to a less presence of phenolic hydroxyl groups due to the esterification process.

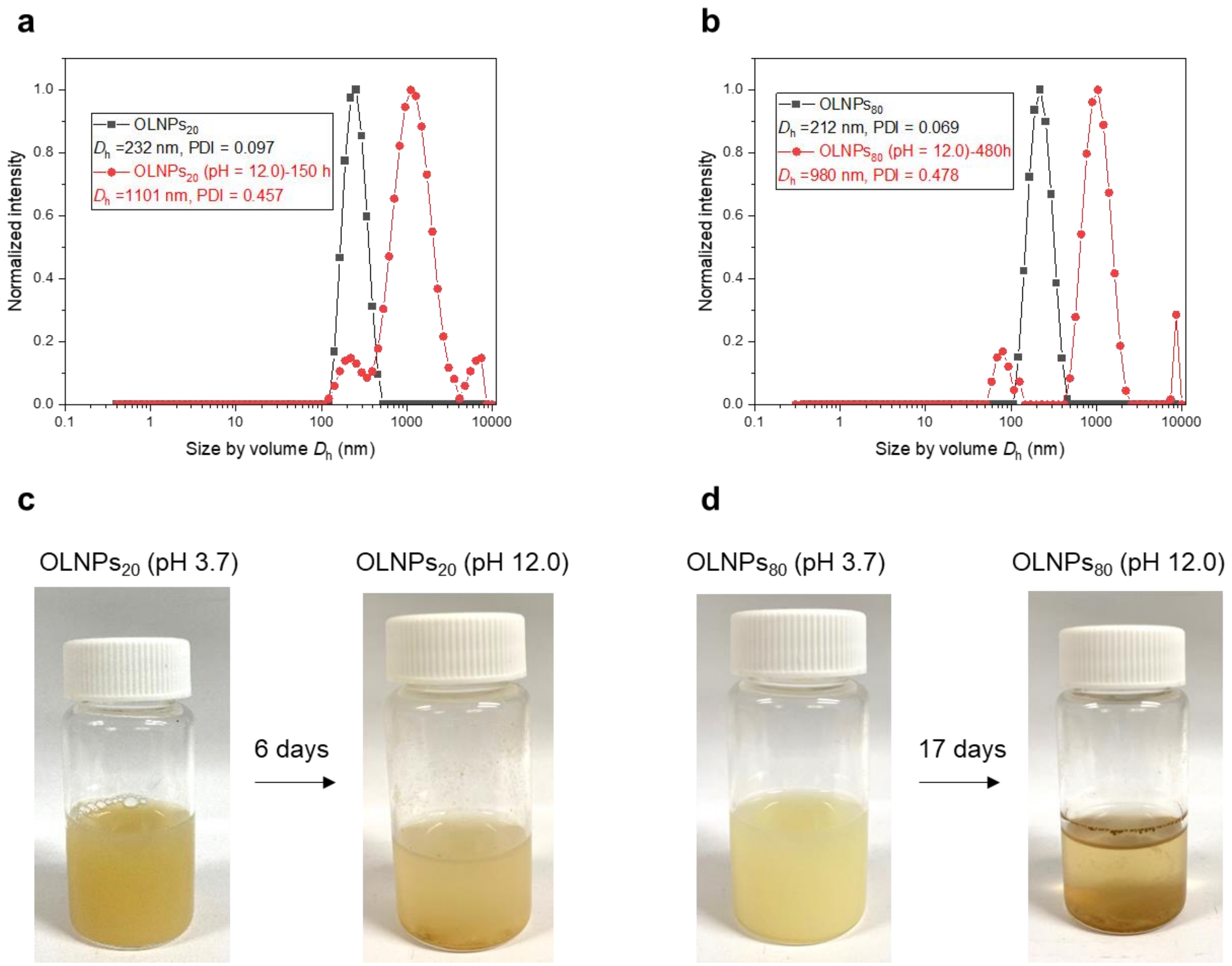

Figure S10. Evolution of particle size for (a) $OLNPs_{20}$ and (b) $OLNPs_{80}$ at pH 12.0 over time. Digital images of the (c) $OLNPs_{20}$ and (d) $OLNPs_{80}$ before and after the aggregation process.

Figure S11 shows the increase in PDI values along with time after the exposure of LNPs and OLNPs to basic conditions (pH = 12.0) as a consequence of particle agglomeration within time.

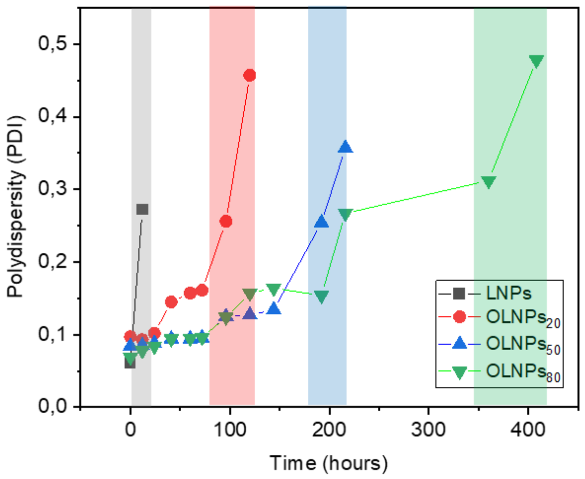

Figure S11. Evolution of polydispersity values for LNPs and OLNPs at pH 12.0. The colored dashed sections indicate the time-dependent aggregation/dissolution for different particles

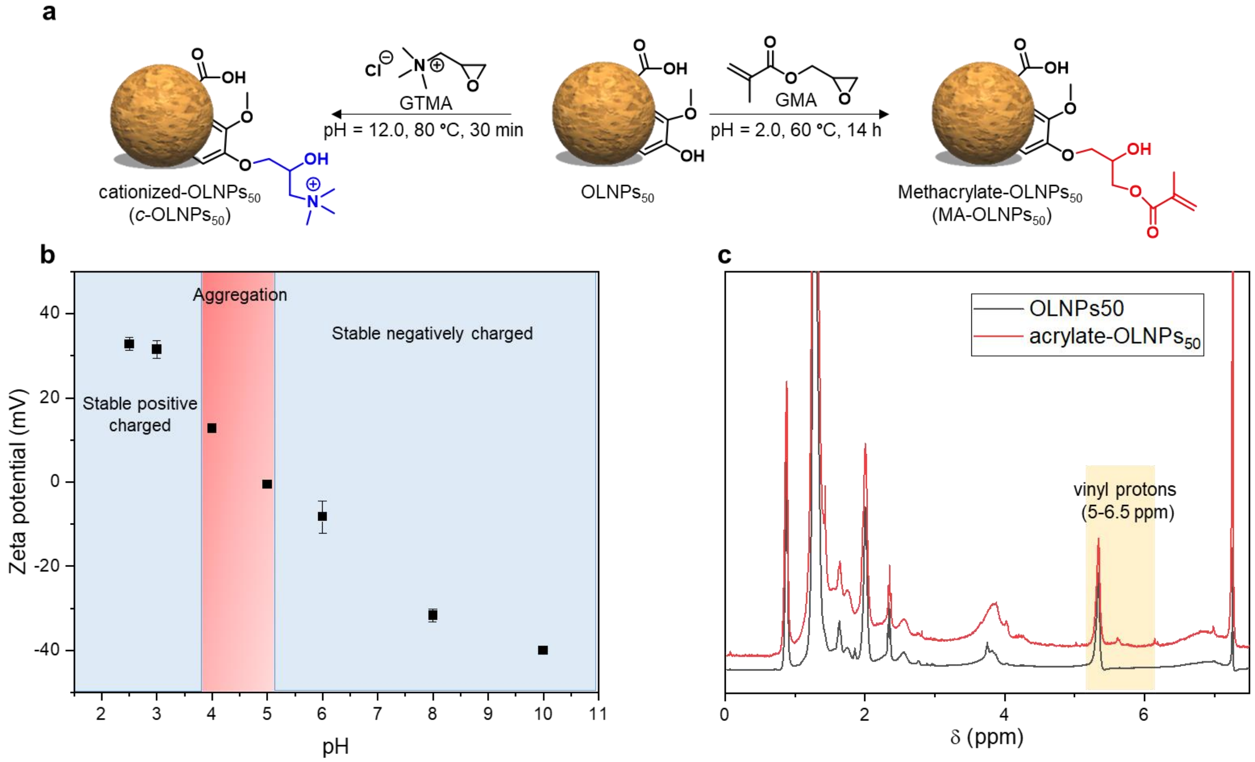

Figure S12. Surface covalent chemical functionalization of $OLNPs_{50}$: (a) left: base-catalyzed ring-opening of GTMA under basic conditions (pH 12.0), right: acid-catalyzed ring-opening reaction of GMA under acidic conditions (pH 2.0). In both cases, $OLNPs_{50}$ was used as a nucleophile for oxirane ring-opening. (b) Zeta potential (mV) of the *c*-OLNPs50 as a function of pH. The dashed sections in blue

indicate stable colloidal dispersion, while the dashed sections in red indicate unstable colloidal dispersion due to the aggregation process. (c) ^{1}H NMR spectra before (black) and after (red) methacrylation of OLNPs50 via ring-opening of GMA. The yellow colored dashed section highlights the presence of vinyl protons after the functionalization.

Figure S13 shows insignificant differences in the particle size between the pristine $OLNPs_{50}$ and the functionalized $OLNPs_{50}$ (c-$OLNPs_{50}$) at different pH values and surface charges.

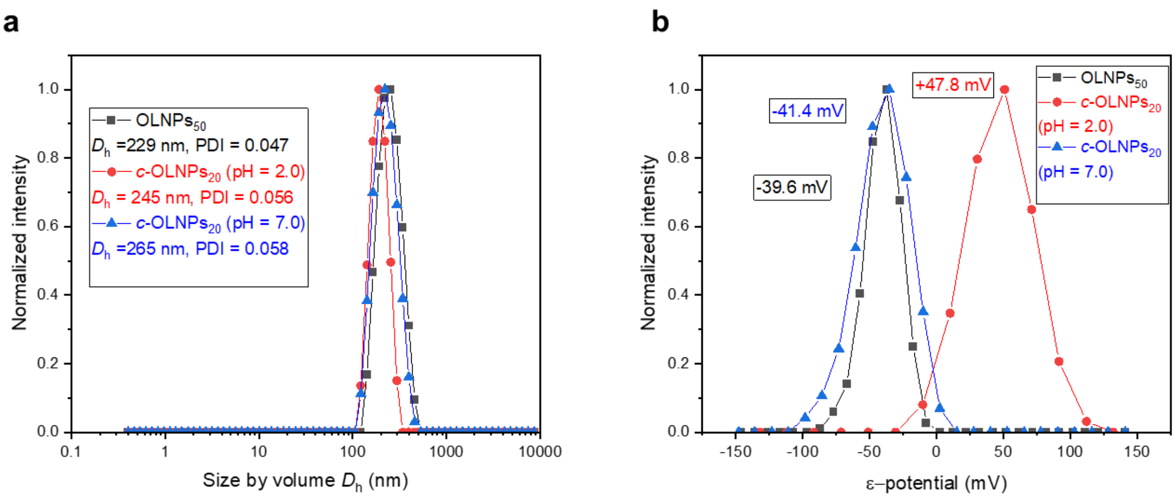

Figure S13. Evolution of (a) particle size and (b) zeta potential for c-$OLNPs_{50}$ at different pH values.

Figure S14 shows the instability of the MA-$OLNPs_{50}$ with the absence of curing step by leaching visualization of the coating after water immersion over time.

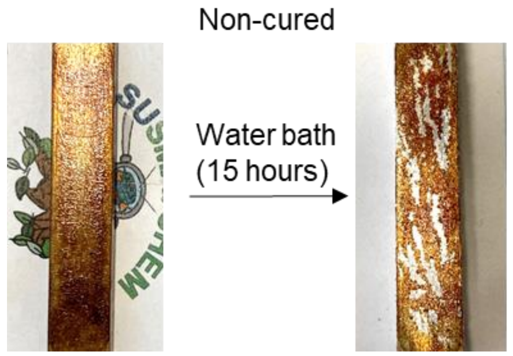

Figure S14. Digital images before and after the exposure of the non-cured Al-coated MA-$OLNPs_{50}$ specimen to saline water (5% NaCl) for 15 hours.

Figure S15 shows the thermal behavior of pristine Kraft lignin used to produce lignin-oleic esters (Lig-Ol) and Lig-Ol_{50} (precursor of MA-$OLNPs_{50}$).

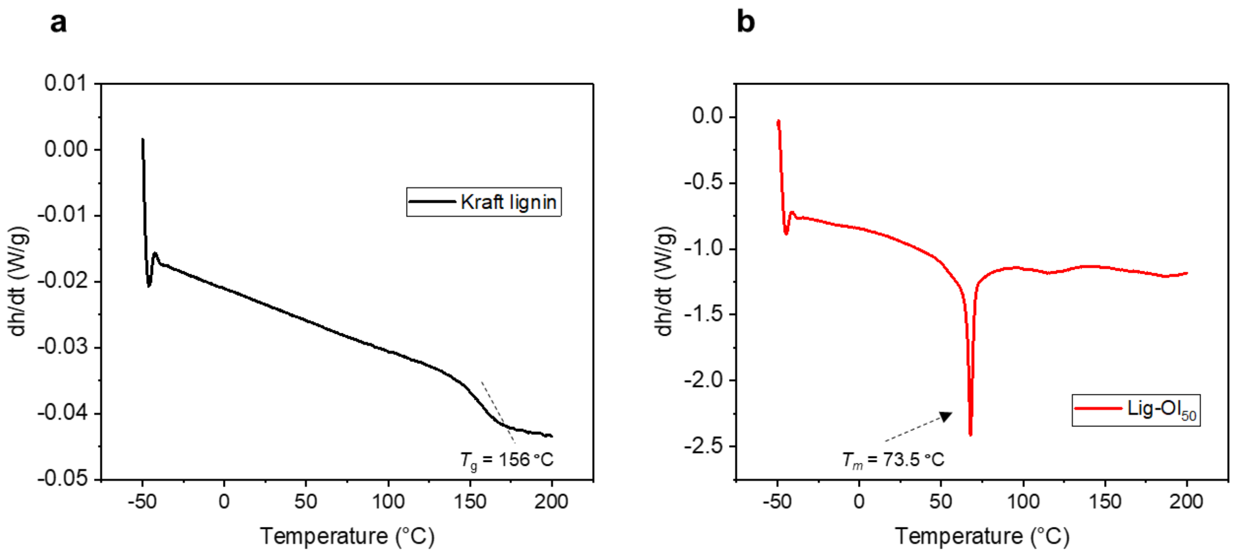

Figure S15. DSC curves for (a) Kraft lignin and (b) Lig-Ol_{50}

Table S3 shows the comparison between the inhibition efficiency (%) for lignin-based and other green anticorrosion coatings for aluminum. As can be seen, the inhibition efficiency (IE,) percentage value obtained from the potentiodynamic polarization experiment demonstrates excellent corrosion resistance after 16 hours at 5% NaCl, outperforming that reported by De Haro et al.[9] for a lignin-based coating under similar conditions, as well as those reported for other green-based corrosion inhibitors.

Table S3. Inhibition efficiency (%) for lignin-based and other green anticorrosion coating for aluminum.

E_{corr} values (V *vs* Ag/AgCl)[1]		j_{corr} values (A.cm^2)		Inhibition efficiency (*IE*%)	Conditions	Ref.
Al_{ref}	Al_{inh}	Al_{ref}	Al_{inh}			
–0.72	–0.79	2.14 x 10^{-6}	1.45 x 10^{-7}	93.2%	Pure Al<br>3.5% NaCl<br>+ 1000 ppm $LaCl_3$	1
–1.2 (*vs* SCE)	–1.3 (*vs* SCE)	5.2 x 10^{-5}	3.0 x 10^{-6}	94%	AA 7075<br>3.5% NaCl 25<br>+ 1000 ppm CCR<br>*C. chinensis* root extract	2
–0.78 (*vs* SCE)	–0.74 (*vs* SCE)	2.09 x 10^{-5}	6.61 x 10^{-7}	96.8%	AA 1050<br>3.5% NaCl + 8 mL.L^{-1}<br>Garlic extract	3
–0.71	–0.686	12.9 x 10^{-6}	3.15 x 10^{-6}	76%	Aluminium<br>0.6 M NaCl<br>+ 50 ppm *Acanthocereus tetragonus* extract	4
–0.770 (*vs* SCE)	–0.868 (*vs* SCE)	1 x 10^{-4}	8.7 x 10^{-6}	90.2%	AA5754<br>3 % NaCl<br>+ 50 ppm *Laurus nobilis* extract	5
–0.765	–0.785	7.49 x 10^{-4}	1.5 x 10^{-5}	98.1%	Aluminium (n.d.)<br>0.1M HCl<br>RGO 0.1g.L^{-1} + 10 μM Pc**2**	6
–1.12	–1.13	2.6 x 10^{-5}	4.6 x 10^{-7}	98.2%[2]	AA 6011 + SiE 8020 coating<br>3.5% NaCl	7
–0.811	–0.602	5 x 10^{-6}	1 x 10^{-7}	98%[2]	AA 6061-T6 + Ce based coating[3]<br>3 % NaCl	8
–0.78	–0.85	4.6 x 10^{-8}	5.05 x 10^{-10}	98.9%[2]	Aluminium<br>5% NaCl<br>CLF-s/HT coating<br>Lignin-based	9
–0.74	–0.8	1.35 x 10^{-4}	2.54 x 10^{-7}	99.8%	Aluminium<br>5% NaCl<br>Lignin-based coating	This work

[1] Unless otherwise stated
[2] Values not given in reference, and calculated according to the equation below from Polarization data
[3] Coating formed from $CeCl_3 \cdot 7H_2O$ and H_2O_2

Calculation of efficiency

$$IE\% = 100 \times \frac{jcorr_{ref} - jcorr_{inh}}{jcorr_{ref}}$$

Where IE% is inhibition efficiency and is dimensionless, $jcorr_{ref}$ and $jcorr_{inh}$ represent, respectively, the current density of unprotected and protected substrates.

References for lignin-based and other green anticorrosion coatings:

[1] A. K. Mishra, R. Balasubramaniam, *Mater. Chem. Phys.* **2007**, *103*, 385–393.

[2] W. Liu, A. Singh, Y. Lin, E. E. Ebenso, G. Tianhan, C. Ren. *C.* **2014**; Vol. 9.

[3] N. Hajsafari, Z. Razaghi, S. H. Tabaian, *J. Mol. Liq.* **2021**, *336*, 116386.

[4] H. G. Méndez Figueroa, R. Galvan-Martinez, A. Y. López- López, J. A. Ramirez-Fernandez, R. Orozco Cruz, *ECS Trans.* **2021**, *101*, 335–342.

[5] J. Halambek, K. Berković, J. Vorkapić-Furač, *J. Mater. Chem. Phys.* **2013**, *137*, 788–795.

[6] N. Nnaji, N. Nwaji, J. Mack, T. Nyokong, *J. Mol. Struct.* **2021**, *1236*, 130279.

[7] S. S. Pathak, A. S. Khanna, *Prog. Org. Coatings* **2009**, *65*, 288–294.

[8] B. Valdez, S. Kiyota, M. Stoytcheva, R. Zlatev, J. M. Bastidas, *Corros. Sci.* **2014**, *87*, 141–149.

[9] J. Carlos De Haro, L. Magagnin, S. Turri, G. Griffini, *ACS Sustain. Chem. Eng.* **2019**, *7*, 6213–6222.

Figure S16 shows the effective adsorption of MB at different concentrations of *c*-OLNPs$_{50}$. The aggregation process starts at 40 mg/g MB: *c*-OLNPs$_{50}$ due to the neutralization of the surface charge.

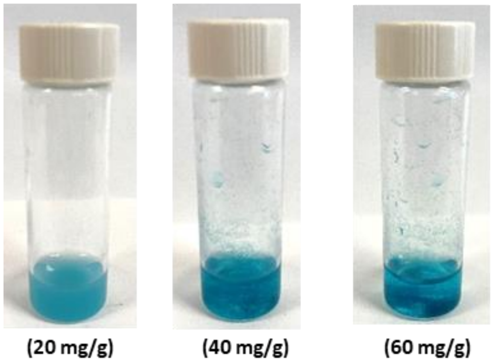

Figure S16. Representative digital images taken at a concentration ratio of MB with respect to *c*-OLNPs$_{50}$.

Table S4 shows the comparison between the adsorption performance vs time for different lignin-based adsorbents towards MB as cationic dye.

Table S4. Adsorption performance of different lignin-based adsorbents towards MB as cationic dye.

Lignin-adsorbent	Maximum capacity (mg/g)	Dye removal efficiency (%)	Time[a]	Ref.
c-OLNPs$_{50}$ (this work)	60	85.6	10 min	-
Aminated lignin	502.7	95	24 h	1
Lignin-sulfonated porous carbon	621.7	92.3	36 h	2
Lignin calcium mesoporous	803.9	72.3	300 min	3
Enzymatic hydrolysis lignin (EHL)	431.1	98.4	180 min	4
Lignin-magnetic particles	211.4	80.5	90 min	5
Alkali lignin	121.2	90.5	72 h	6
Acetic acid lignin	63.3	95.7	72 h	7
Lignin hollow magnetic nanoparticles	31.2	90.3	100 min	8
Organosolv lignin	40.0	85.4	250 min	9
Chitosan-lignin composite	36.25	79.3	75 h	10

[a] Time used to evaluate the dye removal efficiency.

References for lignin-based adsorbents:

[1] X. Meng, B. Scheidemantle, M. Li, Y-Y. Wang, X. Zhao, M. Toro-González, P. Singh, Y. Pu, C. E. Wyman, S. Ozcan, C. M. Cai, A. J. Ragauskas, *ACS Omega*, **2020**, *5*, 2865-2877.

[2] S. Zhu, J. Xu, Y. Kuang, Z. Cheng, Q. Wu, J. Xie, B. Wang, W. Gao, J. Zeng, J. Li, K. Chen, *Ind. Crops Prod.* **2021**, *159*, 113071.

[3] K. Dai, Z. Wang, X. Peng, J. Wu, P. Yang, M. Li, C. Tang, W. Zhang, H. Ying, *ACS Omega*, **2021**, *6*, 816–826.

[4] W. Sui, T. Pang, G. Wang, C. Liu, A. Mahmud, Parvez, C. Si, C. Li, *Molecules*, **2020**, *25*, 2603.

[5] X. Li, Y. He, H. Sui, L. He, *Nanomaterials*, **2018**, 8, 162.

[6] Q. Feng, H. Cheng, J. Li, P. Wang, Y. Xie, *Bioresources*, **2014**, *9*, 3602-3612.

[7] Q. Feng, H. Cheng, F. Chen, X. Zhou, Y. Xie, *J. Wood Chem. Technol.*, **2016**, *36*, 173-181.

[8] Y. Li, M. Wu, B. Wang, Y. Wu, M. Ma, X. Zhang, *ACS Sustainable Chem. Eng.* **2016**, *4*, 5523-5532.

[9] S. Zhang, Z. Wang, Y. Zhang, H. Pan, L. Tao, *Procedia Environ. Sci.* **2016**, *31*, 3– 11.

[10] A. B. Albadarin, M. N. Collins, M. Naushad, S. Shirazian, G. Walker, C. Mangwandi, *Chem. Eng. J.* **2017**, *307*, 264– 272.

Figure S17 shows the effect of the electrolyte addition on the zeta potential measurements.

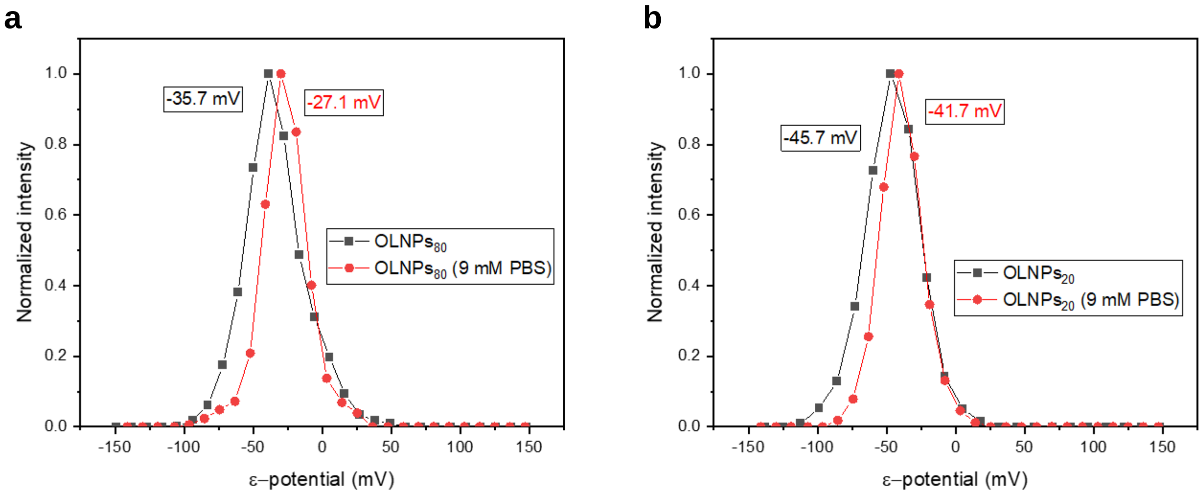

Figure S17. (a) Evolution of zeta potential in (a) $OLNPs_{80}$ and (b) $OLNPs_{20}$ before and after the addition of PBS as an electrolyte.
